# Modulatory Effects of* Astragalus* Polysaccharides on T-Cell Polarization in Mice with Polymicrobial Sepsis

**DOI:** 10.1155/2015/826319

**Published:** 2015-11-26

**Authors:** Yu-Chen Hou, Jin-Ming Wu, Ming-Yang Wang, Ming-Hsun Wu, Kuen-Yuan Chen, Sung-Ling Yeh, Ming-Tsan Lin

**Affiliations:** ^1^Department of Surgery, National Taiwan University Hospital, Taipei 100, Taiwan; ^2^School of Nutrition and Health Sciences, Taipei Medical University, Taipei 110, Taiwan; ^3^Department of Medical Education and Bioethics, College of Medicine, National Taiwan University, Taipei 100, Taiwan

## Abstract

*Background.* This study evaluated the impact of different doses of* Astragalus* polysaccharides (APS) on the functional status and phenotype of T cells during polymicrobial sepsis.* Methods.* On day 1 after cecal ligation and puncture, mice were treated with either saline, 100 (A100), 200 (A200), or 400 mg APS/kg body weight (BW) (A400) by an intraperitoneal injection daily for 4 days. All mice were sacrificed 5 days after the operation.* Results.* APS treatment reversed the sepsis-induced decrement in the T helper (Th) cell population, and the percentage of activated Th cells also increased in the spleen and Peyer's patches. APS administration downregulated the percentages of circulating Th2 cells and regulatory T cells (Treg), and the percentage of Th17 cells in blood was upregulated in the A400 group. Weight loss and kidney injury were attenuated in the A100 and A200 groups but not in the A400 group at the end of the study.* Conclusions.* Treatments with 100 and 200 mg APS/kg BW reduced Treg populations and elicited a more-balanced Th1/Th2 response that consequently attenuated immunosuppression in polymicrobial sepsis. High-dose APS administration led to excessive responses of Th17 cells which may have adverse effects in sepsis-induced organ injury.

## 1. Introduction

Sepsis is a characteristic set of systemic inflammatory responses to bacterial infection. Despite effective treatments with antibiotics and fluid resuscitation, morbidity and mortality from sepsis still remain high in intensive care units [1]. Sepsis activates both pro- and anti-inflammatory immune responses and causes disturbance of the immune system, characterized by a net response of initial hyperinflammation which then enters a persistent immunosuppressive phase [2]. Organ dysfunction caused by the overwhelming inflammation is the most lethal complication of sepsis [3]. Sepsis-induced immunosuppression results in failure to control primary and secondary hospital-acquired infections [4]. Balancing pro- and anti-inflammatory responses has therefore become a potential therapeutic approach for sepsis [2].

Sepsis causes a marked apoptosis-induced depletion of lymphocytes, leading to immunosuppression [5, 6]. The prolonged duration of sepsis enhances the development of T-cell exhaustion which is correlated with nosocomial infections and mortality in septic patients [7]. CD4^+^ T cells, including T helper (Th) cells and regulatory T cells (Treg), play important roles in immune homeostasis during sepsis [8]. Th cells have been characterized into Th1, Th2, and Th17 cell subsets according to the types of cytokines excreted after stimulation. Th1 and Th17 cells protect against pathogen infections by, respectively, promoting the killing ability of macrophages and neutrophils. Th2 cells are considered to be a less protective subset during sepsis due to their enhancement of humoral immunity and inhibition of classical inflammation. Treg are implicated in immunosuppressive properties of T cells and innate immune cells [9]. An increased percentage of circulating Treg were found in septic patients [10], and excessive Treg contribute to lymphocyte anergy in sepsis [11].

The dried root of* Astragalus membranaceus* is thought to tone the vital energy [12], and it has been used as a health-promoting herb for centuries in Asia. Modern research revealed that the active constituents of* Astragalus* include polysaccharides, saponins, flavonoids, amino acids, and trace elements [13].* Astragalus* polysaccharide (APS), the major component obtained from water extraction, was demonstrated to be the pharmacological component that acts as an immunopotentiator [14, 15] and showed suppressive effects on Treg in burned mice with bacterial infections [16]. Also, APS was found to promote a shifting of splenic CD4^+^ T cells from a Th2 to a Th1 cytokine-producing profile in an in vitro study [17]. However, the modulatory effects of APS on T-cell polarization in polymicrobial sepsis remain unclear. Therefore, we investigated the functional status and phenotype of T cells from the circulation and lymphoid organs to evaluate the impacts of different doses of APS administered to control immune homeostasis during sepsis.

## 2. Materials and Methods

### 2.1. Animals

C57Bl/6J male mice at 6~8-week-old and weighing 19~21 g at the beginning of the experiment were used in this study. Mice were purchased from the National Laboratory Animal Center (Taipei, Taiwan) and were housed in a conventional animal facility. All mice were given free access to water and laboratory chow throughout the study. This study was carried out in Taipei Medical University. Animal care and experimental procedures were reviewed and approved by the Institutional Animal Care and Use Committee of Taipei Medical University (approval number LAC-101-0284). All animal experiments were carried out according to the approved protocols. Humane endpoints were considered in this experiment. Mice would be euthanized when showing signs associated with a moribund state, including unconsciousness with no response to external stimuli, intractable seizures, labored breathing, cyanosis, inability to ambulate, and inability to eat or drink.

### 2.2. Experimental Design

After 1 week of acclimation, mice were randomly assigned to receive either a sham operation (*n* = 10) or cecal ligation and puncture (CLP) (*n* = 44). The CLP surgery was used to induce polymicrobial sepsis. A combination of ketamine (80 mg/kg) and xylazine (10 mg/kg) via intraperitoneal (i.p.) injection was used as the anesthetic and analgesic agents. Briefly, under anesthesia, the cecum was exposed, and cecal ligation was performed at approximately 50% of the length of the cecum with 3-0 silk. The distal cecum was then punctured twice with a 22-gauge needle, and a small amount of feces was squeezed out through the perforations. After replacing the cecum back into the abdominal cavity, the musculature and skin were, respectively, closed using 3-0 silk sutures. Sham-operated mice were handled in the same manner except that no ligation or puncture of the cecum was performed. All mice were treated with the antibiotic Ertapenem (INVANZ, Merck, Whitehouse Station, NJ, USA) at a dose of 75 mg/kg body weight (BW) via i.p. injection. Antibiotic treatments were begun 6 h after surgery, and an injection was given every 24 h until day 2 after surgery.

**Figure 1:**
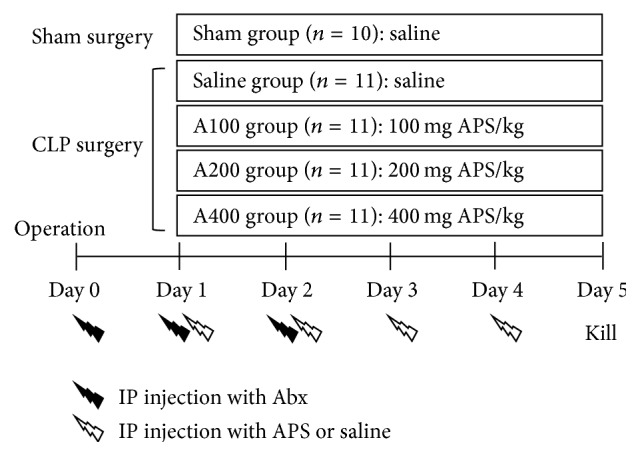
Schematic diagram of the experimental procedure. Abx: antibiotics. APS:* Astragalus* polysaccharides.

On day 1 after surgery, CLP-operated mice were randomly divided into 4 groups (*n* = 11 in each group) as follows: sepsis with normal saline treatment (saline group), sepsis with 100 mg APS/kg BW treatment (A100 group), sepsis with 200 mg APS/kg BW treatment (A200 group), and sepsis with 400 mg APS/kg BW treatment (A400 group). Septic mice received an i.p. injection of APS reconstituted with normal saline daily for 4 days, while mice in the sham and saline groups were given an equivalent volume of normal saline. A flow diagram of the experimental procedures is shown in Figure 1. The APS used in this study were a sterile injectable extract of* Astragalus membranaceus* (PhytoHealth, Taipei, Taiwan). The APS consisted of *α*-1,4(1,6) glucan, arabinose-galactose polysaccharide, rhamnose-galacturonic acid polysaccharide, and arabinose-galactose protein polysaccharide with molecular weights of 20,000~60,000 [17].

BWs and survival rates were recorded daily during the experimental period. On day 5 after the sham or CLP surgery, all mice were killed under anesthesia by cardiac puncture, and fresh blood was collected in heparinized tubes. Peyer's patches (PPs) and the spleen from each animal were removed, and the spleen was cut in half. One-half of a spleen was saved for a flow cytometric analysis, and the other half was stored at −80°C for further measurements. To obtain intestinal lavage fluid (ILF), 5 cm of the small bowel proximal to the ileocecal valve, corresponding to the ileum, was excised and cut open longitudinally. The specimen was immersed in 1 mL of ice-cold phosphate-buffered saline with a protease inhibitor cocktail (Sigma, St. Louis, MO, USA) for 10 min. The immersion fluids with luminal contents were collected and centrifuged for 10 min at 300 ×g. Supernatants were transferred to microfuge tubes and stored at −80°C for further analysis.

### 2.3. Lymphocyte Characterization

Blood, spleens, and PPs were used to analyze lymphocyte populations by flow cytometry. Cell suspensions from PPs and spleens were obtained by passing the tissues through a nylon cell strainer with a 40 *μ*m pore size (BD Biosciences, San Jose, CA, USA) in RPMI1640 medium (Biological Industries, Kibbutz Beit Haemek, Israel). After lysing erythrocytes and centrifugation at 300 ×g for 10 min, pelleted cells were suspended in staining buffer. Whole-blood samples and cell suspensions from spleens and PPs were split into 100 *μ*L aliquots and incubated with PerCP-conjugated anti-CD45 (Clone 30-F11, Biolegend, San Diego, CA, USA), APC-conjugated anti-CD3*ε* (Clone 145-2C11, eBioscience, San Diego, CA, USA), PE-conjugated anti-CD19 (Clone 6D5, Biolegend), FITC-conjugated anti-CD4 (Clone GK1.5, eBioscience), and Pacific blue-conjugated anti-CD8 (Clone 53-6.7, Biolegend) antibodies (Abs). Stained cells were analyzed with a FACS Canto II flow cytometer (BD Biosciences). CD45-positive cells were gated and considered to be leukocytes. Lymphocyte populations were determined as the percentages of T cells (CD3*ε* ^+^CD19^−^) and B cells (CD3*ε* ^−^CD19^+^) among leukocytes. Subpopulations of Th cells and cytotoxic T cells are presented as the percentage of CD4^+^CD8^−^ and CD4^−^CD8^+^ cells among CD3*ε*-expressing cells.

### 2.4. Activation of T Lymphocytes

Expression of the activation marker, CD69, on T lymphocytes was measured by flow cytometry. Aliquots at 100 *μ*L of cell suspensions from spleens and PPs were incubated with PerCP-conjugated anti-CD45, APC-conjugated anti-CD3*ε*, FITC-conjugated anti-CD4, Pacific blue-conjugated anti-CD8, and PE-conjugated anti-CD69 (Biolegend) Abs. Stained cells were run through the flow cytometer. CD45-positive leukocytes were gated, and T-cell subsets were determined as described above. Activated T lymphocytes were evaluated by the expression of CD69 of T-cell subpopulations.

### 2.5. Subsets of CD4-Positive T Cells in Blood

To determine the phenotypes of Th cells, 100 *μ*L aliquots of blood were incubated with a Pacific blue-conjugated anti-CD4 (BD Biosciences) Ab, fixed, and then permeated for intracellular cytokine staining. The following Abs were used for intracellular cytokine staining: Alexa Fluor 488-conjugated anti-interleukin- (IL-) 4 (Biolegend), APC-conjugated anti-interferon- (IFN-) *γ* (BD Biosciences), and PE-conjugated anti-IL-17A (Biolegend) Abs. To analyze Treg, 100 *μ*L aliquots of blood were incubated with Pacific blue-conjugated anti-CD4 and APC-conjugated anti-CD25 (eBioscience) Abs. After incubation for 30 min, leukocytes were fixed and permeated with Foxp3 staining buffer (eBioscience). PE-conjugated anti-Foxp3 (Biolegend) Abs were then added for the intracellular staining of Foxp3.

After staining, red blood cells were lysed, and leukocytes were analyzed by flow cytometry. CD4-positive lymphocytes were gated on the basis of low forward and side scatter. Phenotypes of Th cells are presented as percentages of Th-associated cytokine-expressing cells in CD4-positive lymphocytes. Treg populations are presented as a percentage of CD25/Foxp3 double-positive cells in gated CD4-expressing lymphocytes.

### 2.6. Real-Time Quantitative Polymerase Chain Reaction (qPCR)

RNA extraction from spleens was prepared with the TRIzol reagent (Invitrogen, Carlsbad, CA, USA), and complementary (c)DNA was synthesized from 1 *μ*g of total RNA using a cDNA synthesis kit (Fermentas, Glen Burnie, MD, USA) according to the manufacturers' instructions. Expressions of cytokine genes in spleens were analyzed using the ABI 7300 qPCR System (Applied Biosystems, Foster City, CA, USA). Amplification was performed in a total volume of 25 *μ*L containing 5 *μ*L of 1/100 diluted cDNA, 100 nM of each primer, and 1X SYBR green master mix reagent (Applied Biosystems) using the thermocycling protocol recommended by the PCR system. Primer sequences were as follows: mouse IFN-*γ* (5′-ATGAACGCTACACACTGCATC-3′ and 5′-CCATCCTTTTGCCAGTTCCTC-3′), IL-4 (5′-ACAGGAGAAGGGACGCCAT-3′ and 5′-GAAGCCCTACAGACGAGCTCA-3′), IL-17A (5′-AGCAAGAGATCCTGGTCCTGAA-3′ and 5′-CATCTTCTCGACCCTGAAAGTGA-3′), Foxp3 (5′-AGCGAGTGTCCCTGCTCTCCC-3′ and 5′-CTTCTGTCTGGAGTGGCTGGGTGT-3′), IL-6 (5′-GGGACTGATGCTGGTGACAA-3′ and 5′-ACAGGTCTGTTGGGAGTGGT-3′), IL-2 (5′-AACCTGAAACTCCCCAGGAT-3′ and 5′-TCATCGAATTGGCACTCAAA-3′), transforming growth factor (TGF)-*β* (5′-GCCCTGGATACCAACTATTGCTT-3′ and 5′-AGTTGGCATGGTAGCCCTTG-3′), and *β*-actin (5′-ACCCACACTGTGCCCATCTAC-3′ and 5′-TCGGTGAGGATCTTCATGAGGTA-3′). All samples were analyzed in triplicate, and multiples of change in messenger (m)RNA were calculated by the equation 2^−ΔΔCt^ (ΔCt indicates the difference in threshold cycles between the test gene and *β*-actin, and ΔΔCt indicates the difference in ΔCt between the CLP and sham groups).

### 2.7. Immunoglobulin A (IgA) Quantification in ILF

Luminal IgA was determined by an enzyme-linked immunosorbent assay kit with the corresponding capture antibody for IgA (ICL, Portland, OR, USA). Procedures followed the manufacturer's instructions. The amount of IgA was determined by detecting the antibody conjugated to horseradish peroxidase.

### 2.8. Histopathology

The kidneys were harvested, fixed with 4% paraformaldehyde, and embedded in paraffin. Specimens were sliced into 5 *μ*m-thick sections and stained with hematoxylin-eosin (H&E) for the histopathological analysis. Digital images at 200x magnification per section were captured with a Zeiss Axiophot light microscope (Carl Zeiss MicroImaging LLC, Thornwood, NY, USA) and a Nikon D1X digital camera (Tokyo, Japan). Five fields per section were examined to determine the morphological changes and lesions. The degree of kidney injury was measured by a semiquantitative scoring system according to Kuruş et al. [18]. The total histological score ranged from 0 to 8, which represented the summed scores of interstitial fibrosis and tubular injury in the renal cortex (Table 1).

### 2.9. Statistical Analysis

Data are presented as the mean ± standard error of the mean (SEM). Statistical analyses were performed using GraphPad Prism 5 software (GraphPad Software, San Diego, CA, USA). Survival rates were evaluated by the log-rank test. Differences among groups were analyzed by a one-way analysis of variance (ANOVA) with Tukey's test. A *p* value of <0.05 was considered statistically significant.

## 3. Results

### 3.1. Survival and BW Loss

**Figure 2:**
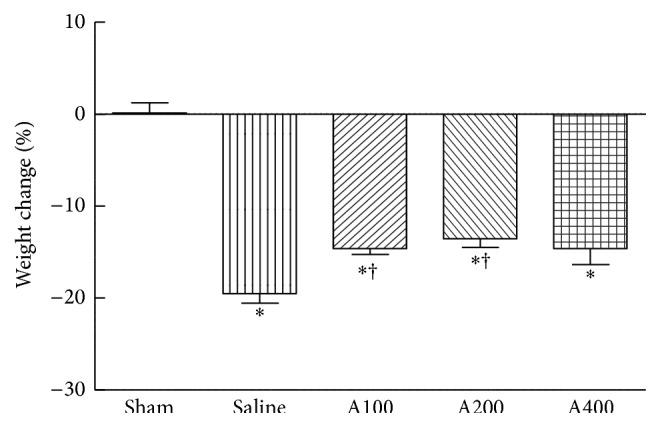
Percentage of body weight changes at the end of the study. Data are presented as the mean ± SEM.  ^*∗*^Significantly different from the sham groups (*p* < 0.05). ^†^Significantly different from the saline group (*p* < 0.05).

No mortality occurred in the sham- or APS-treated groups. In the saline group, one mouse died on day 4 after CLP surgery. The mouse did not show signs associated with a moribund state before death. However, there were no significant differences in survival rates between the sham-operated and CLP mice during the study period. The initial BW among the 5 groups did not differ. BW loss was observed in the sepsis groups compared to the sham group at the end of the study. Weight loss was attenuated in the A100 and A200 groups (Figure 2).

### 3.2. Lymphocyte Populations in Blood and Lymphoid Tissues Were Affected by APS Treatment

Compared to sham-operated mice, percentages of circulating T and B cells decreased in septic mice. Lower percentages of T cells in spleens and B cells in PPs were observed in mice subjected to CLP. Reductions of Th cell subsets in the blood, spleen, and PPs were observed in the saline group (Table 2). APS treatment had no influence on the total circulating and splenic T- and B-cell populations in septic mice. However, higher percentages of T cells were observed in PPs from mice treated with APS. Populations of Th cells in the blood and spleen were maintained by treatments with 200 and 400 mg APS/kg of BW (Table 2). Representative flow cytometry plots are shown in Supplementary Figures  1 and 2 in Supplementary Material available online at http://dx.doi.org/10.1155/2015/826319.

### 3.3. APS Treatment Contributes to Th Cell Activation in the Spleen and PPs

There were no significant differences in activation levels of splenic T cells between the sham and saline groups. The percentage of activated Th cells in PPs from septic mice was lower than that from sham-operated mice (Table 3). APS treatment in septic mice significantly promoted activation of splenic Th cells, and activation levels of Th cells in PPs were maintained in the A200 and A400 groups (Table 3). Representative flow cytometry plots are shown in Supplementary Figure  2.

### 3.4. Circulating Th2 Cells and Treg Are Suppressed by APS Treatment

**Figure 3:**
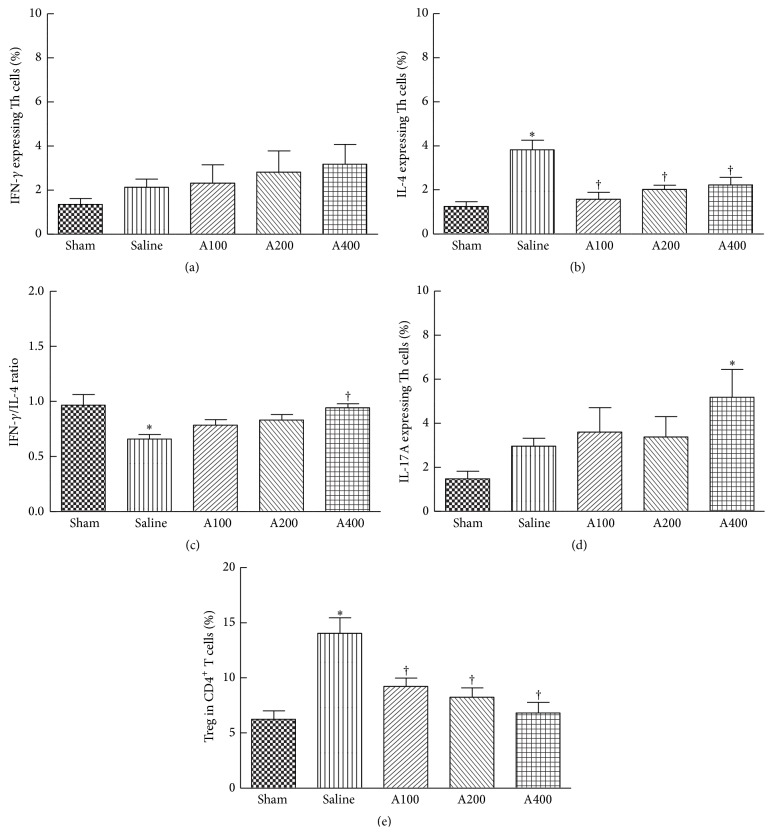
Subpopulations of CD4-positive T cells in the blood. CD4^+^ blood lymphocytes were gated to analyze expression levels of interferon- (IFN-) *γ*, interleukin- (IL-) 4, and IL-17A by flow cytometry (a, b, and d). The ratio of IFN-*γ*- and IL-4-expressing T helper (Th) cells was calculated (c). (e) Percentage of CD4^+^CD25^+^Foxp3^+^ regulatory T cells (Treg) among CD4^+^ lymphocytes. Data are presented as the mean ± SEM.  ^*∗*^Significantly different from the sham group (*p* < 0.05). ^†^Significantly different from the saline group (*p* < 0.05).

There were no significant differences in percentages of IFN-*γ*-producing Th1 cells and IL-17A-expressing Th17 cells between the sham and saline groups (Figures 3(a) and 3(d)). The reduction in the IFN-*γ*/IL4 ratio in the saline group was accompanied by higher percentages of IL-4-expressing Th2 cells (Figures 3(b) and 3(c)). The population of Treg was upregulated in the saline group compared to the sham group (Figure 3(e)). APS treatment in septic mice significantly decreased percentages of IL-4-expressing Th2 cells and the Treg population (Figures 3(b) and 3(e)). Of note, treatments with a high dose of APS elevated the IFN-*γ*/IL-4 ratio (Figure 3(c)) and had promotive effects on IL-17A expression by Th17 cells (Figure 3(d)). Representative flow cytometry plots are shown in Supplementary Figure  3.

### 3.5. Th2- and Treg-Related Genes in the Spleen Are Downregulated by APS Treatment

Compared to the sham group, mice in the saline group had lower expression levels of the IFN-*γ*, IL-17A, IL-6, and IL-2 genes, whereas IL-4 gene expression was upregulated. There were no significant differences in expressions of the Foxp3 and TGF-*β* genes between the sham and saline groups (Table 4). APS treatment had no effects on expressions of the IFN-*γ*, IL-2, and TGF-*β* genes. mRNA levels of IL-4 and Foxp3 were lower in APS-treated groups. Suppression of the IL-17A and IL-6 genes in septic mice was prevented by APS administration. IL-17A and IL-6 gene expressions in the A400 group were higher than those in the sham group (Table 4).

### 3.6. APS Treatments Increase Intestinal IgA Concentrations

**Figure 4:**
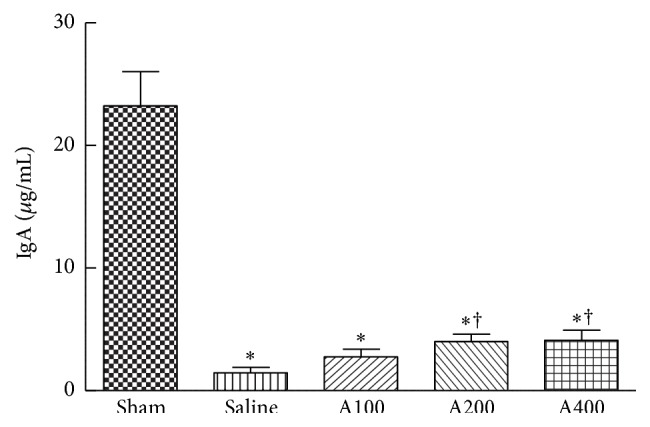
IgA concentration in intestinal lavage fluid. Data are presented as the mean ± SEM.  ^*∗*^Significantly different from the sham groups (*p* < 0.05). ^†^Significantly different from the saline group (*p* < 0.05).

Compared to the sham group, luminal IgA levels gradually decreased in septic mice. Higher IgA concentrations were observed in the A200 and A400 groups than in the saline group (Figure 4).

### 3.7. Histopathological Aspects of the Kidneys

**Figure 5:**
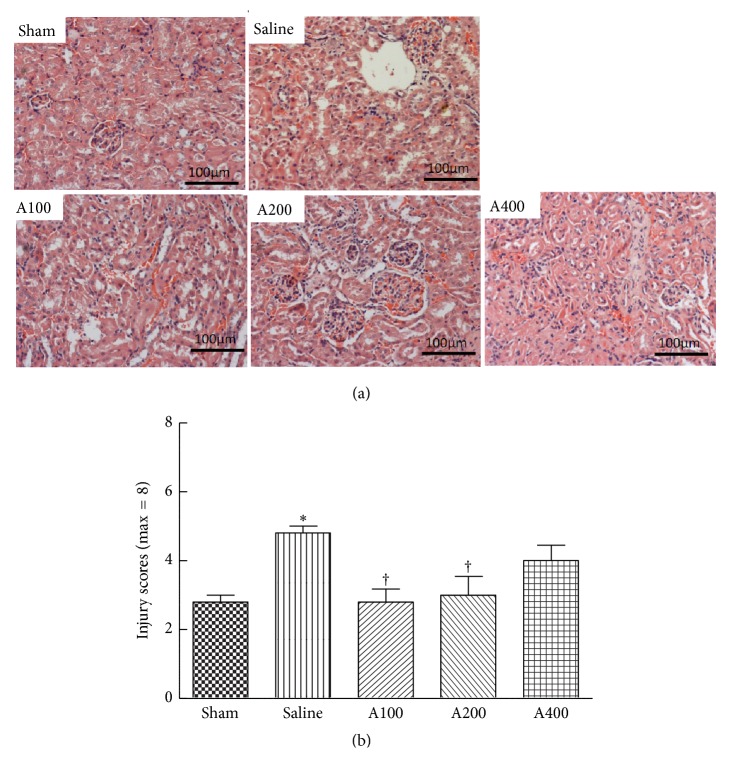
Histopathology of the kidney. Representative histological images are shown at 200x magnification. (a) Kidney tissues stained with hematoxylin and eosin. (b) Histological scores of kidney injury are presented as the mean ± SEM, which were determined as described in Section 2.  ^*∗*^Significantly different from the sham group (*p* < 0.05). ^†^Significantly different from the saline group (*p* < 0.05).

Morphological changes and semiquantitative scores of H&E-stained kidney tissues are shown in Figure 5. Dilated renal tubules and swollen tubular cells were observed in septic mice. Minor kidney injuries were observed in the A100 and A200 groups (Figure 5(b)). Thickened basement membranes of renal tubules were found in mice treated with the highest dose of APS (Figure 5(a)).

## 4. Discussion

*Astragalus membranaceus*, with a long history of use in Chinese herbalism, has been mentioned as an immunomodulator, which was found to stimulate macrophage activity [19] and to reverse the immunosuppression seen in lung cancer patients by inhibiting Th2 polarization [20]. Among the active constituents, APS has been most widely investigated for their immunopotentiating ability, including activating mouse macrophages and stimulating the proliferation of murine splenocytes and human lymphocytes [15, 19]. In this study, we evaluated the outcomes on day 5 after CLP in order to investigate the effects of APS on the regulatory roles of adaptive immune responses during sepsis. Our results show that APS treatments prevented the sepsis-induced decrement of the Th cell population, inhibited Th2 and Treg polarization, and potentiated Th cell activation, which was correlated with attenuating postseptic immunosuppression. The APS dosages used in this study were previously proven to be effective in suppressing Treg in mice under an infectious condition [16], and the low dose of APS (100 mg/kg BW) was equivalent to the clinical dose used to relieve cancer-related fatigue based on the body surface area normalization method [21, 22].

The CLP model is widely used in sepsis research. CLP induced lymphocyte apoptosis, which mimics human sepsis [23]. Mortality in the CLP model is affected by the length of the cecum ligated, the needle size used for perforation, the number of punctures, and antibiotic treatments [24]. In the present study, low mortality was observed in septic mice, which was due to administration of antibiotics. According to a previous study performed in our lab, without antibiotic treatment, the mortality rate of the CLP model used in this study was approximately 45% (unpublished data). Mice were given antibiotics after CLP surgery to imitate a common medical treatment in septic patients, which is more clinically relevant.

Previous studies showed that sepsis results in significant decrements in T lymphocytes and CD4 cell numbers and impairment of T-cell functions [8]. Sepsis-induced apoptosis causes depletion of immune cells leading to immunosuppression [2], and apoptosis of immune cells occurs in lymphoid tissues and gut-associated lymphoid tissues [25]. In this study, we analyzed lymphocytes from the blood, spleen, and PPs, because lymphocytes from the systemic circulation and lymphoid organs are important in regulating the homeostasis of immune function during sepsis. The spleen performs the important function of removing blood-borne pathogens from the circulation. Naïve T cells are activated by circulating antigens in the spleen and differentiate into different effector subsets. Intestinal PPs are essential for mucosal adaptive immunity. Our results indicated that sepsis caused decrements in circulating T and B lymphocytes, reductions of Th cell populations in the blood and lymphoid tissues, and lower Th cell activation in PPs. These findings were compatible with previous reports that sepsis results in lymphocyte depletion and impairment of T-cell function. APS administration reversed sepsis-induced decrements in Th cell populations in the blood and spleen at the higher dose of APS. Also, the percentage of activated Th cells in the spleen and PPs increased when APS was administered. These results indicated that APS prevent depletion of Th cells and enhance activation of Th cells in sepsis.

Th cells and Treg play important roles in the proper development of cellular and humoral immune responses during sepsis. Sepsis-induced abnormalities of CD4 T-cell subpopulations were reported, including polarization to the Th2 subset and increments of Treg populations [2]. Th1 cells produce IFN-*γ* which activates macrophages to kill intracellular microorganisms, whereas Th2 cells secrete IL-4 which enhances the B-cell response and alternative activation of macrophages to promote tissue repair [8]. IFN-*γ* and IL-4 also, respectively, act in an autocrine loop on differentiating Th1 and Th2 cells. The shift from a proinflammatory Th1 phenotype to an anti-inflammatory Th2 phenotype is regulated by mitogen-activated protein kinases (MAPKs). A study by Song et al. [26] reported that induction of p38 MAPK activation in splenic lymphocytes contributes to the immunosuppression seen in septic mice. In the present study, the percentage of IL-4-expressing Th cells in the blood was higher and the IFN-*γ*/IL-4 ratio was lower in the saline group, indicating that Th cells had shifted toward a Th2-type response. Splenic cytokine mRNA expressions were consistent with these results that Th1-related cytokines were lower and Th2 cytokines were higher than those of the sham group. APS administration downregulated circulating Th2 cells, inhibited the Th phenotype toward the Th2 response in the blood, and suppressed splenic IL-4 mRNA expression. We also observed that the sepsis-induced decrement in intestinal IgA was partially reversed in the A200 and A400 groups, suggesting that although APS administration inhibited Th2 polarization, the function of IgA secretion was not impaired. Our results suggested that a more balanced Th1/Th2 response occurred during sepsis when APS was provided. A previous study indicated that APS induced the differentiation of splenic dendritic cells and further modulated the immune function of Th cells with a shift from Th2 to Th1 [17].

Th17 cells, the major source of IL-17A, facilitate neutrophil recruitment and promote clearance of extracellular pathogens by neutrophils [27]. However, the importance of Th17 cell effector functions in sepsis is still under investigation. Flierl et al. [28] indicated that plasma IL-17A increased after CLP surgery, which was mainly derived from *γδ*T cells, and IL-17A promoted high levels of proinflammatory cytokines, resulting in negative outcomes in experimental sepsis. Treg are involved in suppressing excessive T-cell responses, and Foxp3 is a key transcription factor in Treg development and function [9]. Treg are more resistant to sepsis-induced apoptosis than are other effector CD4 T cells [29], which explains the increased percentage of circulating Treg in patients with sepsis [10]. Our results indicated that the percentage of IL-17A-expressing Th cells in the blood was not affected by sepsis, although IL-17A mRNA expression was downregulated in the spleen. Since Th17 cells are not the only source of IL-17A [27], further studies are required to clarify the roles of IL-17A-producing cells in sepsis. A higher blood Treg percentage was also observed in the saline group, which is compatible with a previous report [10].

In this study, declines in IL-17A and IL-6 mRNA expressions in spleen were prevented by APS administration. Treatment with the high dose of APS even upregulated transcription levels of splenic IL-17A and IL-6 mRNA and the population of blood Th17 cells. IL-6 has counter-regulatory effects in modulating Treg and Th17 differentiation from naive T cells. IL-6 promotes the differentiation of Th17 cells together with TGF-*β* [30]. However, Treg differentiation is inhibited by IL-6 [31], which could explain the findings of lower Foxp3 mRNA expression and circulating Treg frequencies in the APS-treated groups. The Treg-suppressing effects of APS were demonstrated in different animal models [16, 32]. In a murine model of burns with bacterial infection, APS was found to suppress the expression of Foxp3 by CD4^+^CD25^+^ Treg and to activate Th1 cell-mediated immunity [16]. Those authors concluded that the influence of APS on Treg was possibly partially mediated by binding the Toll-like receptor 4 on the surface of Treg. Our results suggested that modulation of cytokine profiles by APS also plays important roles in Th and Treg polarization.

Multiple organ failure is a critical complication of sepsis, and sepsis-induced renal dysfunction is associated with the high mortality rate [33]. In order to understand the impact of APS on organ injury, morphological changes and injury scores of kidney tissues were analyzed. The histological findings showed that sepsis resulted in damage to tubular cells, but the damage to the kidneys was less severe in the septic groups with dosages of 100 and 200 mg APS/kg of BW. Since APS administration leads to a more balanced Th/Treg response, this change may subsequently improve sepsis-induced tissue injury. However, kidney injury was not attenuated with APS administration at a dosage of 400 mg APS/kg of BW, which should be due to excessive polarization toward the proinflammatory Th1 and Th17 lineages. Previous studies have indicated that both Th1 and Th17 cells are capable of inducing renal inflammation and injury [34, 35].

In summary, this study showed that APS treatment at dosages of 100 and 200 mg/kg of BW increased Th cell percentages in the circulation and lymphoid tissues, decreased the percentage of Treg, and inhibited the polarization of blood CD4^+^ T cells toward a Th2 response that may consequently attenuate kidney injury in antibiotic-treated septic mice. However, high-dose APS administration may excessively reverse the dysregulated Th/Treg response and have adverse effects in sepsis-induced organ injury. We conclude that APS is an immunopotentiator and proper dosage of APS may be a potential adjuvant treatment for sepsis-induced immunosuppression.

## Supplementary Material

The data presented here is the gating strategy and representative plots for flow cytometry analysis. Supplementary Figure 1 shows the gating strategy of identifying T and B lymphocytes whereas Supplementary Figure 2 shows the gating strategy for definition of T cell subpopulations. Representative plots for analysis of blood Th cells and Treg are shown in Supplementary Figure 3.

## Figures and Tables

**Table 1:** The histological scoring system of kidney injury.

Feature	Score	Description
Interstitial fibrosis	0	Normal interstitium and tubules
	1	Mild interstitial thickening between the tubules
	2	Moderate interstitial thickening between the tubules
	3	Severe interstitial thickening between the tubules

Tubular injury^*∗*^	0	No tubular injury
	1	<10% of tubules injured
	2	10%~25% of tubules injured
	3	26%~50% of tubules injured
	4	51%~75% of tubules injured
	5	>75% of tubules injured

^*∗*^Tubular injury was defined as tubular dilation, tubular atrophy, vacuolization, sloughing of tubular epithelial cells, or thickening of the tubular basement membrane.

**Table 2:** Lymphocyte populations (%).

	Sham	Saline	A100	A200	A400
Blood					
T cells	17.4 ± 0.8	13.1 ± 1.3^*∗*^	12.9 ± 0.8^*∗*^	13.0 ± 1.0^*∗*^	11.7 ± 0.8^*∗*^
Th cells	58.4 ± 1.0	51.4 ± 1.6^*∗*^	51.8 ± 1.5^*∗*^	53.4 ± 1.4	53.7 ± 1.4
Tc cells	32.9 ± 2.8	33.7 ± 3.3	36.0 ± 3.7	36.8 ± 2.8	35.1 ± 3.1
B cells	49.8 ± 1.6	31.1 ± 1.4^*∗*^	30.2 ± 2.6^*∗*^	25.6 ± 2.7^*∗*^	22.3 ± 3.0^*∗*^
Spleen					
T cells	24.2 ± 0.6	20.4 ± 1.0^*∗*^	19.7 ± 0.6^*∗*^	18.9 ± 0.7^*∗*^	19.6 ± 0.7^*∗*^
Th cells	47.6 ± 1.3	39.9 ± 1.2^*∗*^	42.5 ± 0.8^*∗*^	43.5 ± 1.0	44.1 ± 0.8
Tc cells	34.4 ± 0.7	36.1 ± 0.7	34.0 ± 0.6	33.3 ± 0.3	34.0 ± 1.1
B cells	47.1 ± 1.4	48.8 ± 1.4	48.9 ± 1.4	50.5 ± 1.4	49.1 ± 1.3
Peyer's patches					
T cells	8.4 ± 0.5	11.7 ± 1.0	15.6 ± 2.4^*∗*^	16.2 ± 1.8^*∗*^	17.4 ± 2.4^*∗*^
Th cells	53.8 ± 3.7	28.4 ± 1.3^*∗*^	27.3 ± 3.2^*∗*^	31.7 ± 4.1^*∗*^	29.1 ± 2.5^*∗*^
Tc cells	16.9 ± 2.0	14.4 ± 1.7	15.0 ± 2.8	16.6 ± 2.5	15.3 ± 2.5
B cells	77.8 ± 1.9	58.1 ± 2.4^*∗*^	52.7 ± 5.6^*∗*^	52.9 ± 5.2^*∗*^	53.6 ± 6.1^*∗*^

CD45-positive cells were gated to determine the population of lymphocytes by flow cytometry. Populations of T and B cells were determined as the percentages of CD3*ε* ^+^CD19^−^ and CD3*ε* ^−^CD19^+^ cells among CD45-positive cells. Percentages of T helper (Th) and cytotoxic T (Tc) cells were, respectively, determined by CD4^+^CD8^−^ and CD4^−^CD8^+^ cells in CD3*ε*-expressing T lymphocytes. Values are presented as the mean ± SEM. ^*∗*^Significantly different from the sham group (*p* < 0.05).

A100, A200, and A400, respectively, indicate treatment with 100, 200, and 400 mg *Astragalus* polysaccharides/kg of body weight.

**Table 3:** T lymphocyte activation (%).

	Sham	Saline	A100	A200	A400
Spleen					
CD69^+^ Th cells	10.4 ± 0.6	12.4 ± 1.2	14.5 ± 0.9^*∗*^	15.3 ± 0.8^*∗*^	16.6 ± 1.0^*∗*^
CD69^+^ Tc cells	4.6 ± 0.4	7.4 ± 1.4	7.0 ± 0.8	8.1 ± 1.5	8.3 ± 2.1
Peyer's patches					
CD69^+^ Th cells	53.6 ± 2.5	38.3 ± 3.2^*∗*^	37.7 ± 2.5^*∗*^	45.6 ± 2.3	46.0 ± 4.0
CD69^+^ Tc cells	42.8 ± 6.2	45.3 ± 6.8	43.3 ± 6.3	42.5 ± 5.4	46.1 ± 5.7

T helper (Th) and cytotoxic T (Tc) cells were, respectively, identified by CD4^+^CD8^−^ and CD4^−^CD8^+^ cells among CD3*ε*-positive cells using a flow cytometer. Activated T cells were determined by the expression of CD69 on T-cell subsets. Values are presented as the mean ± SEM. ^*∗*^Significantly different from the sham group (*p* < 0.05).

A100, A200, and A400, respectively, indicate treatment with 100, 200, and 400 mg *Astragalus* polysaccharides/kg of body weight.

**Table 4:** Expression of T-cell polarization-related genes in the spleen.

	Sham	Saline	A100	A200	A400
Relative mRNA ratio					
IFN-*γ*	1.07 ± 0.06	0.33 ± 0.03^*∗*^	0.42 ± 0.10^*∗*^	0.46 ± 0.03^*∗*^	0.42 ± 0.08^*∗*^
IL-4	1.06 ± 0.04	2.52 ± 0.20^*∗*^	1.55 ± 0.20^†^	1.52 ± 0.17^†^	1.13 ± 0.26^†^
IL-17A	1.06 ± 0.06	0.43 ± 0.07^*∗*^	0.71 ± 0.07	0.72 ± 0.12	0.90 ± 0.17^†^
Foxp3	1.00 ± 0.07	0.78 ± 0.07	0.53 ± 0.06^*∗*^	0.54 ± 0.02^*∗*^	0.55 ± 0.11^*∗*^
IL-6	1.00 ± 0.02	0.45 ± 0.03^*∗*^	0.92 ± 0.12	0.96 ± 0.12	1.33 ± 0.23^†^
IL-2	1.02 ± 0.10	0.53 ± 0.08^*∗*^	0.57 ± 0.10^*∗*^	0.65 ± 0.05^*∗*^	0.64 ± 0.08^*∗*^
TGF-*β*	1.00 ± 0.09	0.79 ± 0.07	0.92 ± 0.09	0.83 ± 0.04	0.98 ± 0.08

The relative amount of mRNA was calculated using the comparative Ct method, and mRNA expression of sham-operated mice was used as a calibrator. Data are presented as the mean ± SEM. ^*∗*^Significantly different from the sham group (*p* < 0.05). ^†^Significantly different from the saline group (*p* < 0.05).

A100, A200, and A400, respectively, indicate treatment with 100, 200, and 400 mg *Astragalus* polysaccharides/kg of body weight.

IFN: interferon; IL: interleukin; TGF: transforming growth factor.
